# Supplementary material for: Investigation of the N-Glycosylation of the SARS-CoV-2 S Protein Contained in VLPs Produced in Nicotiana benthamiana
Source: Molecules. 2022 Aug 11;27(16):5119. doi: 10.3390/molecules27165119 (PMC9412417; doi:10.3390/molecules27165119)
Supplement: Supplementary file 1 [file molecules-27-05119-s001.zip › molecules-1822663-supplementary.pdf]

Supplementary Materials

MFVFLVLLPLVSSQCVNLTTRTQLPPAYTNSFTRGVYYPDKVFRSSVLHSTQDLFLPFFSNVTWFHAIHVSGTNGTKRFDNPVLPFN
DGVYFASTEKSNIIRGWIFGTTLDSKTQSLLIVNNATNVVIKVCEFQFCNDPFLGVYYHKNNKSWMESEFRVYSSANNCTFEYVSQP
FLMDLEGKQGNFKNLREFVFKNIDGYFKIYSKHTPINLVRDLPQGFSALEPLVDLPIGINITRFQTLLALHRSYLTPGDSSSGWTAGAA
AYYVGYLQPRTFLLKYNENGTITDAVDCALDPLSETKCTLKSFTVEKGIYQTSNFRVQPTESIVRFPNITNLCPFGEVFNATRFASVYA
WNRKRISNCVADYSVLYNSASFSTFKCYGVSPTKLNDLCFTNVYADSFVIRGDEVRQIAPGQTGKIADYNYKLPDDFTGCVIAWNSN
NLDSKVGGNYNYLYRLFRKSNLKPFERDISTEIYQAGSTPCNGVEGFNCYFPLQSYGFQPTNGVGYQPYRVVVLSFELLHAPATVCG
PKKSTNLVKNKCVNFNFNGLTGTGVLTESNKKFLPFQQFGRDIADTTDAVRDPQTLEILDITPCSFGGVSVITPGTNTSNQVAVLYQD
VNCTEVPVAIHADQLTPTWRVYSTGSNVFQTRAGCLIGAEHVNNSYECDIPIGAGICASYQTQTNSPRRARSVASQSIIAYTMSLGA
ENSVAYSNNSIAIPTNFTISVTTEILPVSMTKTSVDCTMYICGDSTECSNLLLQYGSFCTQLNRALTGIAVEQDKNTQEVFAQVKQIYK
TPPIKDFGGFNFSQILPDPSKPSKRSFIEDLLFNKVTLADAGFIKQYGDCLGDIAARDLICAQKFNGLTVLPPLLTDEMIAQYTSALLAG
TITSGWTFGAGAALQIPFAMQMAYRFNGIGVTQNVLYENQKLIANQFNSAIGKIQDSLSSTASALGKLQDVVNQNAQALNTLVKQ
LSSNFGAISSVLNDILSRLDKVEAEVQIDRLITGRLQSLQTYVTQQLIRAAEIRASANLAATKMSECVLGQSKRVDFCGKGYHLMSFP
QSAPHGVVFLHVTYVPAQEKNFTTAPAICHDGKAHFPREGVFVSNGTHWFVTQRNFYEPQIITTDNTFVSGNCDVVIGIVNNTVY
DPLQPELDSFKEELDKYFKNHTSPDVDLGDISGINASVVNIQKEIDRLNEVAKNLNESLIDLQELGKYEQYIKWPWYIWLGFIAGLIAI
VMVTIMLCCMTSCCSCLKGCCSCGSCCKFDEDDSEPVLKGVKLHYT

Figure S1. Amino acids sequence of the SARS-CoV-2 S protein produced recombinantly in tobacco plants. The 22 predicted *N*-glycosylation consensus sites are underlined in black.

A)

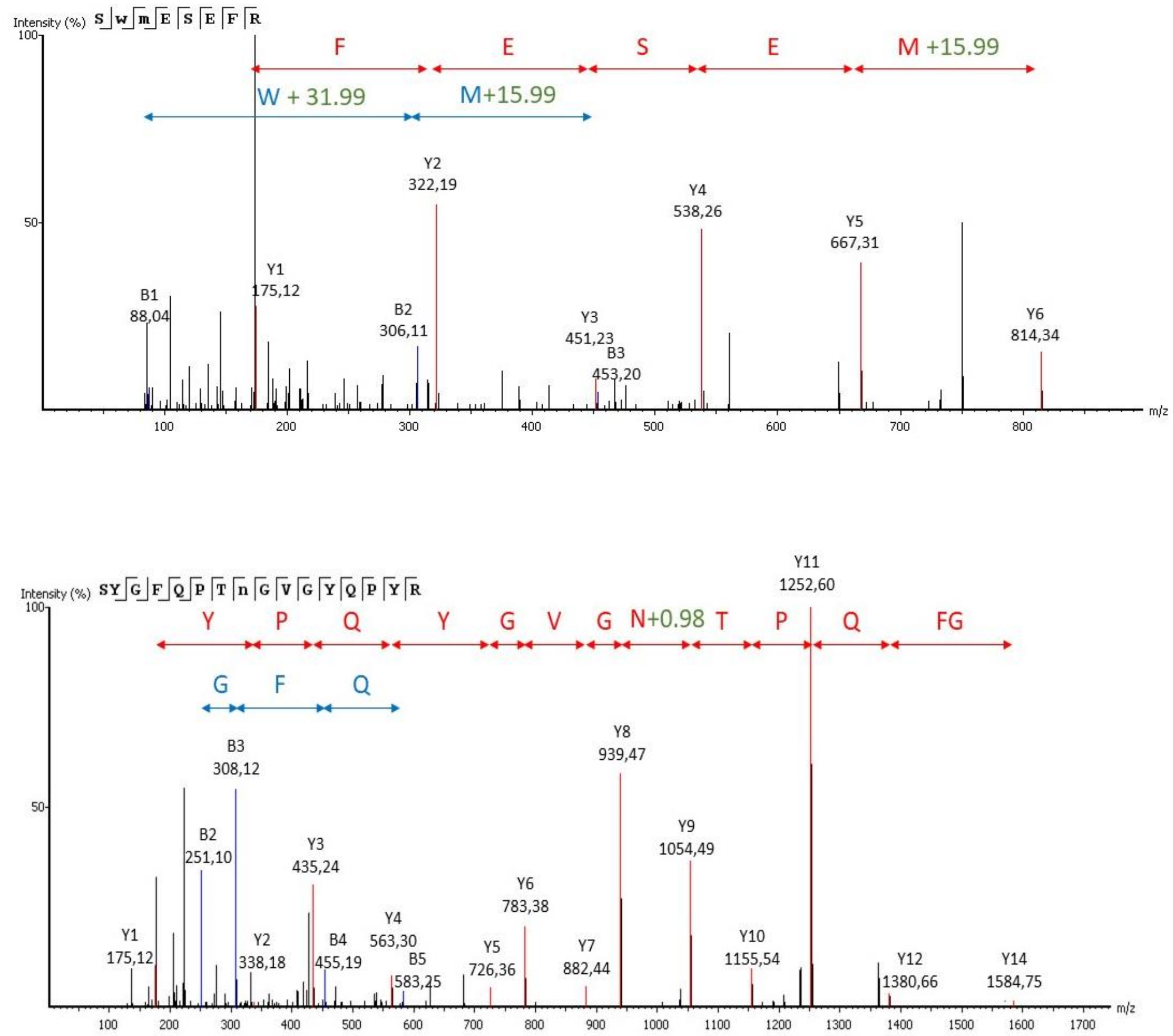

Figure S2. MS/MS spectra of (A) dicharged ion at m/z 560.22 assigned to the peptide S_{151}-R_{158} bearing a dihydroxylation of W_{152} and an oxidation of M_{153}, (B) dicharged ion at m/z 918.42 assigned to the peptide S_{494}-R_{509} bearing a deamidation of N_{501}.

Table S1. Excel file presenting the distribution of *N*-linked glycans on the glycosylation sites of the SARS-CoV-2 S protein produced in *N. benthamiana*. * See Figure 3 for structure and nomenclature of *N*-glycans. T: trypsin; A: AspN, C: chymotrypsin and G: GluC.

Glycosite	Sequence	Enzymes	Calculated peptide $[M+H]^+$	Observed peptide $[M+H]^+$	MS/MS diagnostic ions	Observed peptide + GlcNAc $[M+H]^+$	Observed gycopeptide $[M+H]^+$	Relative intensity (%)	Glycans*
17	L.VSSQCVNLTTR.T	T + A	1264.63	1264.62	204.09 / 366.14	1467.71	2841.22	48.8	Gn2XF
17	L.VSSQCVNLTTR.T	T + A	1264.63	1264.63	204.09 / 366.14 / 512.20	1467.72	2946.24	38.0	LeaMXF
17	L.VSSQCVNLTTR.T	T + A	1264.63	1264.62	204.09 / 366.14 / 512.20	1467.72	3457.45	13.2	Lea2XF
61	F.FSNVTWF.H	C	900.42	900.42	204.09 / 366.14	1103.50	2477.02	100	Gn2XF
74	H.AIHVSGTNGTK.R	T + A	1084.58	1084.58	204.09 / 366.14	1287.66	2458.08	3.2	GnMXF
74	H.AIHVSGTNGTK.R	T + A	1084.58	1084.57	204.09 / 366.12	1287.65	2661.17	25.0	Gn2XF
74	H.AIHVSGTNGTK.R	T + A	1084.58	1084.57	204.09 / 366.14 / 512.20	1287.67	2969.28	47.9	LeaGnXF
74	H.AIHVSGTNGTK.R	T + A	1084.58	1084.58	204.09 / 366.14 / 512.20	1287.66	3277.39	23.9	Lea2XF
122	K.TQSLLIVNNATNVVIK.V	T + A	1727.01	1727.01	204.09 / 366.14	1930.09	3100.52	39.7	GnMXF
122	K.TQSLLIVNNATNVVIK.V	T + A	1727.01	1727.01	204.09 / 366.12	1930.09	3303.6	7.4	Gn2XF
122	K.TQSLLIVNNATNVVIK.V	T + A	1727.01	1727.01	204.09 / 366.14 / 512.20	1930.09	3919.82	52.9	Lea2XF
149	D.PFLGVYYHKNNK.S	T + A	1479.78	1479.78	204.09 / 366.14 / 512.20	1682.86	3364.48	52.6	LeaGnXF
149	D.PFLGVYYHKNNK.S	T + A	1479.78	1479.78	204.09 / 366.14 / 512.20	1682.86	3672.59	47.4	Lea2XF
165	R.VYSSANNCTFEYV.S	T + A	1553.66	1553.70	204.09 / 366.14	1756.80	3130.32	100	Gn2XF
234	V.DLPIGINITR.F	T + A	1111.65	1111.65	204.09 / 366.14	1314.72	2339.10	15.3	GnMX
234	V.DLPIGINITR.F	T + A	1111.65	1111.64	204.09 / 366.14	1314.72	2369.10	7.6	GnM4
234	V.DLPIGINITR.F	T + A	1111.65	1111.65	204.09 / 366.14	1314.72	2490.14	20.0	Man-6
234	V.DLPIGINITR.F	T + A	1111.65	1111.64	204.09 / 366.14	1314.73	2501.14	9.2	GnM4X
234	V.DLPIGINITR.F	T + A	1111.65	1111.65	204.09 / 366.14	1314.72	2531.16	14.9	GnM5
234	V.DLPIGINITR.F	T + A	1111.65	1111.65	204.09 / 366.14	1314.72	2652.18	24.4	Man-7
234	V.DLPIGINITR.F	T + A	1111.65	1111.64	204.09 / 366.14	1314.72	2814.23	8.6	Man-8
282	K.YNENGTIT.D	T + A	911.41	911.41	204.09 / 366.14	1114.49	2081.84	44.0	M2XF
282	K.YNENGTIT.D	T + A	911.41	911.41	204.09 / 366.14	1114.50	2487.99	30.3	Gn2XF
282	K.YNENGTIT.D	T + A	911.41	911.41	204.09 / 366.14 / 512.20	1114.49	2796.12	25.7	LeaGnXF
331	E.SIVRFPNITNLCPFGE.V	T + G	1863.94	1863.91	204.09 / 366.14 / 512.20	2066.98	3748.61	100	LeaGnXF
343	E.VFNATR.F	T + G	707.38	707.38	204.09 / 366.14	910.46	2080.88	41.1	GnMXF
343	E.VFNATR.F	T + G	707.38	707.38	204.09 / 366.14	910.46	2137.90	13.7	Gn2X
343	E.VFNATR.F	T + G	707.38	707.39	204.09 / 366.14	910.46	2283.96	26.4	Gn2XF
343	E.VFNATR.F	T + G	707.38	707.38	204.09 / 366.14 / 512.20	910.47	2592.08	18.8	LeaGnXF
603	I.TPGTNTSNQVAVLYQ.D	T + A	1592.79	1592.80	204.09 / 366.14	1795.68	2834.04	100	GnMF
616	Q.DVNCTEVPVAIHA.D	T + A	1424.69	1424.69	204.09 / 366.14	1627.77	3001.29	74.5	Gn2XF
616	Q.DVNCTEVPVAIHA.D	T + A	1424.69	1424.69	204.09 / 366.14 / 512.20	1627.77	3309.39	25.5	LeaGnXF
657	R.AGCLIGAEHVNNSYEC.D	T + A	1793.76	1793.76	204.09 / 366.14	1996.85	3167.28	7.9	GnMXF
657	R.AGCLIGAEHVNNSYEC.D	T + A	1793.76	1793.77	204.09 / 366.14	1996.85	3370.35	30.9	Gn2XF
657	R.AGCLIGAEHVNNSYEC.D	T + A	1793.76	1793.76	204.09 / 366.14 / 512.20	1996.85	3678.47	17.5	LeaGnXF
657	R.AGCLIGAEHVNNSYEC.D	T + A	1793.76	1793.77	204.09 / 366.14 / 512.20	1996.85	3986.58	43.7	Lea2XF
709	A.YTMSLGAENSVAYSNNS.I	T + A	1807.78	1807.77	204.09 / 366.14	2010.87	3384.35	44.4	Gn2XF
709	A.YTMSLGAENSVAYSNNS.I	T + A	1807.78	1807.78	204.09 / 366.14 / 512.20	2010.89	3692.48	21.7	LeaGnXF
709	A.YTMSLGAENSVAYSNNS.I	T + A	1807.78	1807.78	204.09 / 366.14 / 512.20	2010.87	4000.58	33.9	Lea2XF
717	S.IAIPTNFTISVTT.E	T + G	1377.76	1377.72	204.09 / 366.14	1580.80	2954.31	40.7	Gn2XF
717	S.IAIPTNFTISVTT.E	T + G	1377.76	1377.74	204.09 / 366.14 / 512.20	1580.81	3059.33	12.7	LeaMXF
717	S.IAIPTNFTISVTT.E	T + G	1377.76	1377.72	204.09 / 366.14 / 512.20	1580.80	3262.43	14.4	LeaGnXF
717	S.IAIPTNFTISVTT.E	T + G	1377.76	1377.72	204.09 / 366.14 / 512.20	1580.80	3570.53	32.2	Lea2XF
801	K.DFGGFNFSQI.L	T + A	1131.51	1131.53	204.09 / 366.14	1334.61	2505.04	10.5	GnMXF
801	K.DFGGFNFSQI.L	T + A	1131.51	1131.52	204.09 / 366.14	1334.60	2708.1	69.9	Gn2XF
801	K.DFGGFNFSQI.L	T + A	1131.51	1131.53	204.09 / 366.14 / 512.20	1334.61	3324.35	19.6	Lea2XF
1074	K.NFTTAPAICHDGK.A	T + A	1431.67	1431.67	204.09 / 366.14	1634.76	2659.11	17.9	GnMX
1074	K.NFTTAPAICHDGK.A	T + A	1431.67	1431.67	204.09 / 366.14	1634.75	2805.16	19.2	GnMXF
1074	K.NFTTAPAICHDGK.A	T + A	1431.67	1431.67	204.09 / 366.14	1634.75	2862.21	25.5	Gn2X
1074	K.NFTTAPAICHDGK.A	T + A	1431.67	1431.67	204.09 / 366.14	1634.75	3008.25	23.2	Gn2XF
1074	K.NFTTAPAICHDGK.A	T + A	1431.67	1431.67	204.09 / 366.14 / 512.20	1634.75	3624.48	14.2	Lea2XF
1098	R.EGVFVSNGTHWFVTQR.N	T + A	1863.91	1863.90	204.09 / 366.14	2066.99	3440.50	29.1	Gn2XF
1098	R.EGVFVSNGTHWFVTQR.N	T + A	1863.91	1863.91	204.09 / 366.14 / 512.20	2067.00	3545.54	19.9	LeaMXF
1098	R.EGVFVSNGTHWFVTQR.N	T + A	1863.91	1863.91	204.09 / 366.14 / 512.20	2066.99	3748.61	24.5	LeaGnXF
1098	R.EGVFVSNGTHWFVTQR.N	T + A	1863.91	1863.91	204.09 / 366.14 / 512.20	2067.03	4056.74	26.5	Lea2XF
1158	K.NHTSPDVDLG.D	T + A	1054.48	1054.48	204.09 / 366.14	1257.56	2631.07	38.1	Gn2XF
1158	K.NHTSPDVDLG.D	T + A	1054.48	1054.48	204.09 / 366.14 / 512.20	1257.57	2939.18	17.4	LeaGnXF
1158	K.NHTSPDVDLG.D	T + A	1054.48	1054.48	204.09 / 366.14 / 512.20	1257.56	3247.31	44.5	Lea2XF
1173	G.DISGINASVVNIQK.E	T + A	1457.79	1457.79	204.09 / 366.14	1660.88	2831.31	19.1	GnMXF
1173	G.DISGINASVVNIQK.E	T + A	1457.79	1457.80	204.09 / 366.14 / 512.20	1660.88	3342.51	56.6	LeaGnXF
1173	G.DISGINASVVNIQK.E	T + A	1457.79	1457.80	204.09 / 366.14 / 512.20	1660.88	3650.63	24.3	Lea2XF
1194	K.NLNESLIDLQELGK.Y	T + G	1585.84	1585.85	204.09 / 366.14	1788.90	3162.43	100	Gn2XF
